# The Impact of Fine Particulate Matter 2.5 on the Cardiovascular System: A Review of the Invisible Killer

**DOI:** 10.3390/nano12152656

**Published:** 2022-08-02

**Authors:** Shaherin Basith, Balachandran Manavalan, Tae Hwan Shin, Chan Bae Park, Wang-Soo Lee, Jaetaek Kim, Gwang Lee

**Affiliations:** 1 Department of Physiology, Ajou University School of Medicine, Suwon 16499, Korea; shaherinb@gmail.com (S.B.); catholicon@ajou.ac.kr (T.H.S.); chanbaepark@ajou.ac.kr (C.B.P.); 2 Computational Biology and Bioinformatics Laboratory, Department of Integrative Biotechnology, College of Biotechnology and Bioengineering, Sungkyunkwan University, Suwon 16419, Korea; bala2022@skku.edu; 3 Department of Internal Medicine, Division of Cardiology, College of Medicine, Chung-Ang University, Seoul 06973, Korea; wslee1227@cau.ac.kr; 4 Department of Internal Medicine, Division of Endocrinology and Metabolism, College of Medicine, Chung-Ang University, Seoul 06973, Korea; 5 Department of Molecular Science and Technology, Ajou University, Suwon 16499, Korea

**Keywords:** air pollution, particulate matter, cardiovascular disease, cardiovascular system, ambient, exposure, mitigation, mitochondria

## Abstract

Air pollution exerts several deleterious effects on the cardiovascular system, with cardiovascular disease (CVD) accounting for 80% of all premature deaths caused by air pollution. Short-term exposure to particulate matter 2.5 (PM_2.5_) leads to acute CVD-associated deaths and nonfatal events, whereas long-term exposure increases CVD-associated risk of death and reduces longevity. Here, we summarize published data illustrating how PM_2.5_ may impact the cardiovascular system to provide information on the mechanisms by which it may contribute to CVDs. We provide an overview of PM_2.5_, its associated health risks, global statistics, mechanistic underpinnings related to mitochondria, and hazardous biological effects. We elaborate on the association between PM_2.5_ exposure and CVD development and examine preventive PM_2.5_ exposure measures and future strategies for combating PM_2.5_-related adverse health effects. The insights gained can provide critical guidelines for preventing pollution-related CVDs through governmental, societal, and personal measures, thereby benefitting humanity and slowing climate change.

## 1. Introduction

Air pollution refers to the release of pollutants into the atmosphere, which causes detrimental effects on living beings. Annually, more than 7 million people worldwide die prematurely due to air pollution, more than any other form of pollution [1,2]. As ranked by the Institute for Health Metrics and Evaluation in 2020, air pollution is the fourth leading cause of mortality among all metabolic and behavioral risk factors [1]. Moreover, recent World Health Organization (WHO) statistics showed that 9 out of 10 people breathe air that has pollution levels exceeding the WHO guidelines; therefore, these people are at an increased risk of noncommunicable diseases (NCDs) such as chronic obstructive pulmonary disease (COPD), cardiac diseases, lung cancer, and stroke. The premature mortality rate due to air pollution is three times greater than that caused by malaria, tuberculosis, and acquired immunodeficiency syndrome together and 15 times higher than that due to wars or other violent causes [3]. The mortality rate associated with air pollution in low- and middle-income countries is 100-fold that of high-income countries. During the past 20 years, heart disease has been the leading cause of death worldwide, accounting for 40–60% of all premature deaths due to air pollution [4,5,6]. Air pollutants have been linked to endothelial dysfunction and vasoconstriction, blood pressure (BP) elevation, prothrombotic and coagulant changes, systemic inflammatory and oxidative stress responses, autonomic dysfunction, arrhythmias, and atherosclerosis [7].

It has been predicted that reducing air pollution to WHO air quality standards would increase life expectancy by 0.6 years [5,8]. However, the levels of air pollutants are continuously increasing in both developing countries and developed countries. This is mainly because the stringent air quality guidelines set by the WHO are far from being met, and many breaches in the regulations have been documented in urban areas [3]. According to the United States Environmental Protection Agency (US EPA), particulate matter (PM)_2.5_ is defined as “fine inhalable particles with diameters of generally ≤2.5 µm” [4]. Small PM (PM_2.5_) is considered the most lethal fraction of air pollution, and it was the fifth leading risk factor for mortality in 2015 [6]. Concentrations of ambient PM_2.5_ are measured in micrograms of PM per cubic meter of air (μg/m^3^). When PM_2.5_ is inhaled, it penetrates deep into the lower respiratory tract and reaches the blood and other organs via translocation through membrane receptors. Owing to the larger surface area of PM_2.5_, adsorption of heavy metals, toxic agents, and organic materials is facilitated, thereby enabling the generation of reactive oxygen species (ROS) in the blood and lungs.

Approximately 18.6 million deaths were attributed to cardiovascular disease (CVD) in 2019 globally, including 957,000 deaths in the U.S. [9]. Although PM_2.5_ is associated with a large number of NCDs, approximately half of these deaths are caused by its cardiovascular effects [4,10]. The detrimental effects of air pollution on the cardiovascular system (CVS) came to prominence in the early 1990s, and a markedly linear relationship between PM_2.5_ levels and CVD-related mortality and morbidity was observed [11,12]. There is accumulating evidence that PM_2.5_ exerts toxic effects on the CVS, with increased risk of arrhythmia, atherosclerosis, hypertension, myocardial infarction (MI), stroke, thrombosis, and heart failure exacerbation within hours to days of exposure in susceptible individuals [4]. Similar to PM_2.5_, black carbon (BC), a common and potent contributor to PM_2.5_ total mass, is also linked with cardiovascular effects [13]. Recently, several epidemiological and experimental studies have reported the adverse effects of air pollution on the heart and its vasculature; however, the underlying biomechanisms in cellular organelles remain largely elusive. Mitochondria are one of the major targets of environmental pollutants because of their involvement in xenobiotic metabolism, including air toxicants [14].

In this review, we present information on the current knowledge gaps between PM_2.5_ and CVD systematically by evaluating literature findings, epidemiological studies, and clinical manifestations. Thus, a better understanding of the detrimental cardiovascular effects associated with exposure to PM_2.5_ may be provided, thereby leading to improved risk assessments and potentially guiding the development of tailored interventions to mitigate those adverse effects. We herein present a review on the composition and sources of PM_2.5_, related biological mechanisms, effects of PM_2.5_ on mitochondria, and epidemiological studies on short- and long-term effects. We also specifically elaborate on the degree of evidence of the association of the effects of PM_2.5_ and various CVDs. Lastly, we discuss several public and personal mitigation strategies related to exposure to air pollution and address future viewpoints.

## 2. Composition and Sources of Particulate Matter

Our body is exposed to many contaminants present in air, and hence, it needs to be protected. PM concentration is a common proxy indicator of air pollution. Inhalable PM includes ultrafine, fine, and coarse particles with aerodynamic diameter <0.1 μm, ≤2.5 μm (PM_2.5_), and 2.5–10 μm (PM_2.5–10_), respectively [4,15,16,17]. Typically, smaller PM fractions exert more effects, as they have a larger reactive surface area and can infiltrate deeply into the pulmonary alveoli and then potentially into the bloodstream. Even though inhaled PM_10_ and PM_2.5_ particles can penetrate through the lungs and enter the bloodstream, PM_2.5_ poses a greater health risk than PM_10_ [18]. Chronic exposure to PM increases the risk of developing cardiovascular and respiratory diseases, as well as lung cancer.

There are multiple and context-specific sources of air pollution. Three types of particles present in PM_2.5_ are as follows: primary particles (elemental carbon), secondary particles (organic aerosols), and nitrate and sulfate particles. Primary PM_2.5_ is directly emitted from both natural sources (aerosolized soil, dust storms, forest fires, pollen, molds, and volcanic eruptions) and anthropogenic activities (agriculture or waste incineration, biomass burning, burning of wood, crustal or road dust, cigarette smoke, cooking, construction, fossil fuel combustion, household heating, industry, mechanical wear, power plants, sea salt, and transportation) [19]. The formation of secondary PM_2.5_ in the atmosphere occurs via the condensation of low-volatility products released through the chemical reactions of organic and inorganic precursors [20]. The chemicals emitted from automobile exhausts or coal combustion react with water vapor in the air and sunlight to form novel particles. Overall, PM_2.5_ includes several elements (black carbon, organic carbon and sulfates), sea salt (sodium and chloride), and metal oxides (aluminum, calcium, iron, potassium, silicone, titanium, and zinc) [21,22]. Notably, PM_2.5_ derived from residual oil combustion and traffic sources is known to exert short-term effects on human health, whereas PM_2.5_ generated from coal combustion is known to exert long-term adverse effects [23].

PM_2.5_ composition varies with regard to the different chemical combinations of particles along with variable factors including regions, climate, and anthropogenic activities. Rural areas have high levels of crustal materials such as silicon and aluminum. Air pollution in urban areas has received the most attention owing to the high population density, greater traffic-related emissions, elevated levels of secondary aerosols (ammonium, nitrates, and sulfates), high combustion levels (elemental and organic carbon), and the increasing urbanization of societies globally. Industrialized areas account for high levels of trace elements including zinc, iron, and palladium [24].

## 3. Biological Pathways Linking PM_2.5_ and CVD

**Figure 1:**
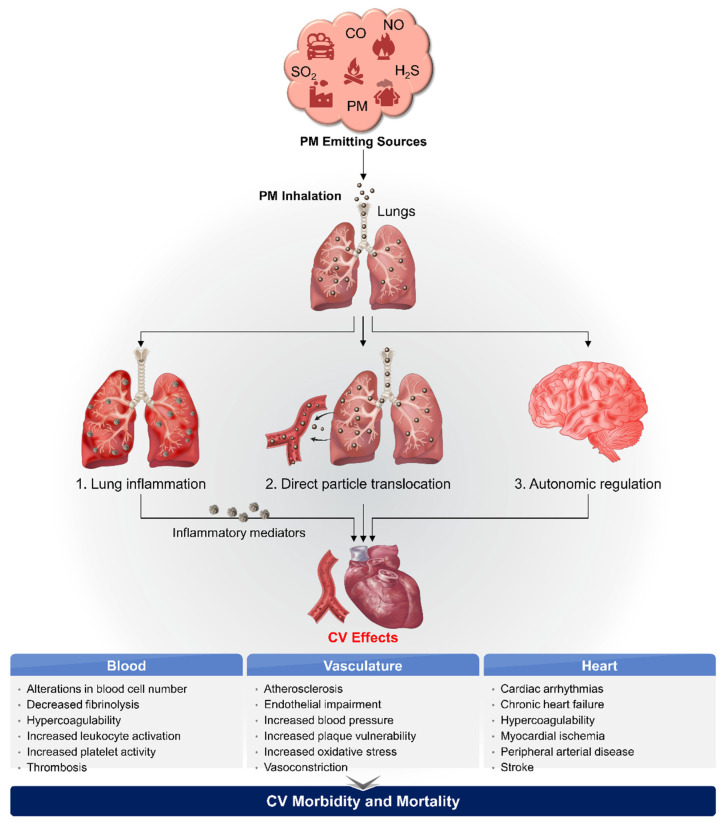
Biological pathways associated with particulate matter (PM) and cardiovascular disease (CVD). Primary signaling pathways through which inhaled PM can induce the incidence of cardiovascular events, eventually leading to cardiovascular morbidity and mortality. Adapted from Ref. [5].

Although a large body of evidence has improved our understanding of the biological mechanisms underlying air pollution-mediated cardiovascular effects, this topic remains to be fully elucidated [4,5,17,25,26]. The primary mechanisms through which PM_2.5_ influences the incidence of cardiovascular events are intricate, multiple, and interdependent. PM inhalation stimulates extrapulmonary effects on the CVS through three biological pathways: (1) oxidative stress and systemic inflammation; (2) direct translocation into systemic circulation; and (3) perturbation of the autonomic nervous system (ANS) (Figure 1).

### 3.1. Oxidative Stress and Systemic Inflammation

Oxidative stress and systemic inflammation are the primary mechanisms by which PM_2.5_ increases the risk of CVDs [22]. Inhaled air pollutants induce oxidative stress in various cell types of the respiratory tract and can be transmitted systematically, contributing to the activation of several effector mechanisms including inflammation [27,28]. Inhaled PM_2.5_ triggers a variety of inflammatory mediators in the lungs, including pro-oxidative (ROS) and pro-inflammatory (tumor necrosis factor, interleukin [IL]-1β, IL-6, interferon-γ, and granulocyte macrophage colony-stimulating factor) initiators, vasoactive hormones (endothelin), and acute-phase reactants (C-reactive protein), which are released into the blood circulation [29,30]. Innate immune receptors, such as toll-like receptors (TLR2/4) and nucleotide oligomerization domain-like receptors, may be activated by a direct or indirect mechanism through secondary mediators, including ROS. Additionally, ion channels, including transient receptor potential (TRP) receptors such as TRPA1 and TRPV1, are activated by oxidative stress caused by combustion or soluble particles [31,32,33].

Activated pulmonary endothelial cells release adhesion molecules, thereby leading to the binding and induction of leukocytes and platelets and resulting in the systemic activation of blood coagulation [34,35]. This finding is supported by reports from previous studies that showed associations between high levels of PM_2.5_ and hypercoagulability markers and increased thrombin formation [35,36]. Furthermore, an ROS-activated pathway is involved in the PM-stimulated pro-inflammatory mechanism, which is linked to atherosclerosis, arrhythmias, MI, and vascular dysfunction [37]. PM_2.5_, along with other gaseous co-pollutants such as ozone has also been shown to increase the effects of PM. It has been reported that when ozone gas levels increase from approximately 30 ppb to 100 ppb, the half-lives of antioxidants are reduced from days to hours, and the half-lives of surfactants are reduced from hours to minutes [38].

### 3.2. Direct Translocation into Systemic Circulation

Due to the small size and large surface area of PM_2.5_, it can cross the pulmonary epithelium and reach the heart and other organs, causing adverse health effects. Preliminary studies substantiate that PM_2.5_ and ultra-fine particles can translocate directly into the pulmonary and systemic circulations [39,40]. Inhaled PM_2.5_ causes pulmonary inflammation that can spread to the circulatory system, resulting in abnormal hemostatic activity [41]. In a number of epidemiological studies, PM_2.5_ exposure has been associated with venous thrombosis and reduced plasma clotting time [42]. Acute PM_2.5_ exposure is linked to increased arterial stiffness [43] and blood pressure [44]. Acute thrombosis such as MI and stroke can be exacerbated by exposure to PM_2.5_ [45].

### 3.3. Perturbation of the Autonomic Nervous System

In normal conditions, the rhythmic activity of the heart is regulated by the activity of autorhythmic cells in the sinoatrial node, which is controlled by the vagus nerve [46]. Acute exposure to PM_2.5_ particles can perturb the ANS and increase the risk of arrhythmia and other cardiovascular events [47]. Heart rate (HR) and HR variability (HRV) are the two main factors associated with cardiac death in patients with heart failure. Controlled exposure studies in humans have shown alterations in BP and HRV [26], while studies in canine and mouse models [48,49] have shown the progression of hypertension upon stimulation of the central sympathetic nervous system by PM_2.5_. Nasal, bronchial, and pulmonary C-nerve fiber subtypes function as an afferent loop through the activation of membrane receptors such as TRPA1, TRPV1, and purinergic P2X channels in response to air pollutants [50,51,52]. Similarly, urinary catecholamines serve as markers of sympathetic activation after prolonged exposure to air pollutants [53].

## 4. PM_2.5_ and Mitochondrial Dysfunction

Mitochondria are promising targets because of their role in the regulation of cell metabolism by the production of ATP, regulation of carbohydrate, lipid, and pyrimidine metabolism, ionic balance maintenance (calcium, copper, and iron homeostasis), regulation of apoptosis, and their function as a hub for several cardioprotective signaling molecules [14]. The mitochondria are also involved in the metabolism of xenobiotics (including environmental toxicants) with the aid of mitochondrial P450s, as they act as a primary source of free radicals upon toxicant exposure [54]. Previous studies have suggested that environmental toxicants can damage mitochondrial DNA (mtDNA) and alter mitochondrial gene expression [55]. Notably, mtDNA lacks the ability to repair DNA; therefore, it is more vulnerable to oxidative damage than nuclear DNA [56]. Mitochondria are considered the primary source of environmental toxicant accumulation owing to their high membrane lipid content. This entails the accumulation of a wide variety of compounds, including PM and polycyclic aromatic hydrocarbons [57].

The mitochondrial inner membrane harbors four multi-subunit electron transport chain (ETC) complexes (complexes I–IV), which are the machinery for generating membrane potential and hence ATP. ETC complexes transport electrons from both NADH and FADH2 to molecular oxygen to produce water, and hydrogen ions are pumped from the matrix towards the mitochondrial intramembrane space, forming the membrane potential and driving ATP synthesis with the aid of ATP synthase. PM_2.5_-exposed rat cardiac fibers and vascular endothelial cells display abnormalities in the mitochondrial membrane ultrastructure, such as swollen and disordered cristae. PM_2.5_ induces aberrant morphologies of mitochondria by dysregulating mitochondrial fission/fusion and size [1,58], indicating the failure of quality control in mitochondrial function [1,59]. Morphological defects in the mitochondrial membrane have a direct impact on the activities of its residents, such as ETC complexes. The PM_2.5_-mediated reduction of ETC complex activity has been described in different cell types. Dysfunctional ETC complexes fail to maintain membrane potential, and reduction in membrane potential and ATP synthesis have been reported [60,61,62]. Opening of the mitochondrial permeability transition pore by PM_2.5_ causes further reduction of membrane potential and ATP synthesis, eventually leading to cell death. Mitochondrial dysfunction and subsequent cell death trigger inflammation in various tissues [63]. Recent evidence suggests that mitochondrial dysfunction plays an important role in PM_2.5_-mediated inflammation responses [64,65,66]. The release of mtDNA and N-formyl peptides from dysfunctional mitochondria acts as a damage-associated molecular pattern and triggers inflammation. Increased ROS production by PM_2.5_ is also directly related to the activity of ETC complexes. Dysfunctional ETC complexes slow down respiration and increase the pool of NADH in mitochondria, which enhances ROS generation [67,68].

Air pollutants mediate CVD by targeting mitochondria through either the inflammation-mediated pathway or the oxidative stress-induced pathway [14]. Mitochondrial membrane alterations mediated by air pollutants can affect the ETC complexes and their dynamic nature. In addition to xenobiotics, metal toxicants (cadmium, lead, manganese, and mercury) and certain chemicals (ethidium bromide, MPP+, and paraquat) accumulate in mitochondria [57,69]. A high number of mitochondria per cell has been reported, which can offset mitochondrial dysfunction or damage. However, the number and consequences differ depending on the type of cells or tissues involved. Because the heart possesses the highest mitochondrial content, even a moderate level of mitochondrial damage may cause adverse effects [14]. An increasing number of studies have shown the significance of mitochondria as a primary ROS source in air pollution-associated pathology. Excess production of mitochondrial ROS is often associated with several CVDs such as atherosclerosis, hypertension, MI, and myocardial ischemia-reperfusion injury [70,71,72]. A key summary of the investigational studies demonstrating the effects of PM_2.5_ on mitochondria in the CVS is shown in Table 1.

## 5. Epidemiological Studies on the Short- and Long-Term Effects of PM_2.5_ in the CVS

**Figure 2:**
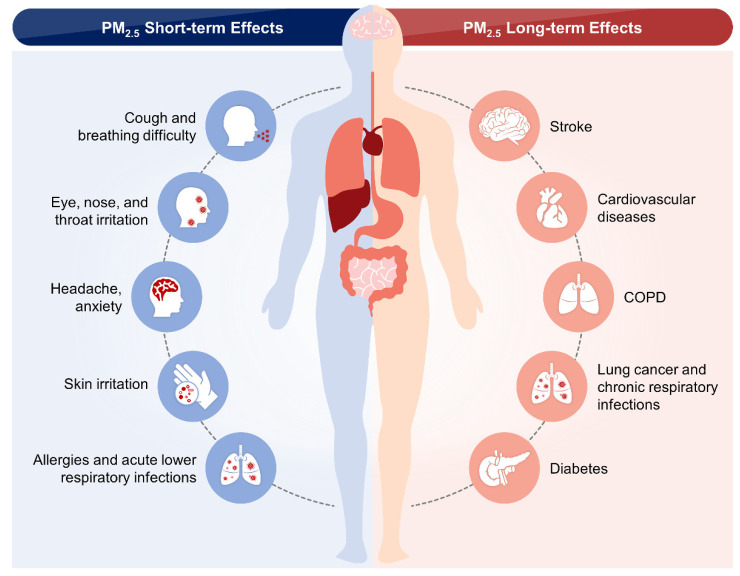
Short- and long-term effects of PM_2.5_ on human health. The inhalation of particulate matter (PM) can irritate the lining of the nasal cavity, thereby inducing runny nose and cough. Inhaled PM can also travel deep down the airways and enter the lungs, thus triggering inflammation and causing shortness of breath, as well as worsening preexisting respiratory diseases such as asthma and chronic obstructive pulmonary disease (COPD). The inflammation can also spread to other parts of the body, leading to the risk of cardiovascular diseases. Lung cancer-related deaths are also related to the adverse effects of PM.

The health of susceptible populations, including children, pregnant women, elderly individuals, and people with chronic diseases, can deteriorate even on low-pollution days. Epidemiological evidence demonstrates that exposure to PM_2.5_ can cause serious health problems [80]. The impact of short-term exposure to air pollutants is temporary and ranges from simple discomfort, including irritation of the eyes, nose, skin, and throat, to breathing difficulties, cough, chest tightness, and wheezing, to more serious health effects such as asthma, COPD, lung and heart problems, frequent hospitalization, pneumonia, and respiratory illnesses [18]. Additionally, short-term exposure can trigger dizziness, headache, and nausea [18]. These health effects can be worsened by prolonged long-term exposure, which damages other body systems, including the neurological, reproductive, and respiratory systems (Figure 2). The long-term effects of PM last for years or are lifelong and can even induce different types of cancers and death [81].

Several epidemiological studies have linked air pollution to several cardiovascular conditions [4], such as cardiac arrhythmia [82], coronary artery disease [83], cerebrovascular disease [84], heart failure [85], peripheral arterial disease (PAD) [86], and venous thromboembolism (VTE) [87]. In 1993, a pioneering study was conducted over a period of 14–16 years to assess the levels of air pollution in six major U.S. cities, where high levels of PM_2.5_ exposure were linked to cardiovascular morbidity and mortality [11]. The American Heart Association writing group reported that short-term PM exposure leads to acute cardiovascular morbidity and mortality, while long-term exposure could shorten life expectancy by a few years [88]. In general, PM_2.5_ exposure leads to a high risk of arrhythmia, heart failure exacerbation, MI, and stroke within hours to days [4]. Numerous epidemiological studies on PM exposure using different experimental designs have been published (Table 2). Table 2 summarizes recent epidemiological studies reported during the years 2017–2021 showing sizeable associations between PM_2.5_ exposure and cardiovascular-related morbidity and mortality. Recently, Zhang et al. [89] conducted a meta-analysis to examine the sex differences linked to IHD and stroke with long-term PM_2.5_ exposure. The study identified that long-term PM_2.5_ exposure in women was associated with an increased risk of IHD (relative risk (RR), 1.21; 95% confidence interval (CI), 1.15–1.27). The additional women-to-men ratio of RR (RRR) was 1.05 (95% CI, 1.02–1.08) per 10 μg/m^3^ increment in PM_2.5_ exposure. Wu et al. [90] conducted a time-series study to estimate the link between short-term PM_2.5_ exposure and CVD-related hospitalizations in Lanzhou, China, and showed that short-term PM_2.5_ exposure increased the hospitalizations for total CVD, especially IHD in male and elderly populations. Another recent open cohort study [91] was conducted to investigate the associations between long-term PM_2.5_ exposure and cardiovascular events as well as CVD-specific mortality among hemodialysis patients in the U.S. A PM_2.5_ level of 1 μg/m^3^ was associated with an increased risk of cardiovascular events (1.02, 95% CI: 1.01, 1.02) and CVD-specific mortality (1.02, 95% CI: 1.02, 1.03). 

Notably, studies on short-term exposure have assessed the cardiovascular consequences of PM_2.5_ based on hourly or daily variations in pollutant concentrations, whereas studies on long-term exposure have investigated annual variations in pollutant concentrations. In general, epidemiological studies have examined blood and urine samples to identify mechanistic markers accountable for cardiovascular changes. However, the relative magnitudes of short- and long-term effects lack clarity owing to the use of different epidemiological methods and exposure errors.

Researchers have found substantial correlations between PM exposure and adverse effects on cardiac autonomic activity [92], electrical instability [93], and myocardial perfusion in panel and controlled-exposure studies [94,95]. HRV, a measure of cardiac function, has been associated with PM exposure, where deleterious changes have been observed [96,97]. In addition to HRV, PM_2.5_ has been associated with changes in cardiac rhythm [98], cardiac ischemia [99], and severe arrhythmia [95]. Furthermore, a meta-analysis reported that a daily increase of 10 μg/m^3^ in PM_2.5_ exposure was linked to a 0.84% increase in cardiovascular-related deaths [100]. In addition to the aforementioned epidemiological studies, several others have also linked PM_2.5_ exposure to the development of cardiovascular conditions.

**Table 2:** Epidemiological studies published in the last five years (2017–2021) investigating the effects of PM_2.5_ on the cardiovascular system.

	Study and Year	Study Name	Study Period	Number of Participants	Age Range of Participants	Country, Region	PM_2.5_, μg/m^3^ (Mean or Range)	Outcome Types
Short-term PM_2.5_ exposure studies	Achilleos et al. 2017 [101]	Meta-analysis (Pubmed and Web of Science)	1996–July 2015	3851 records	All ages	Europe, U.S., West Pacific, Canada, and South America	10	Mortality: Cardiovascular disease (CVD) (0.80% (95% CI: 0.41, 1.20%).
	Newell et al. 2017 [102]	Meta-analysis (PubMed, Web of Science, Embase, LILACS, Global Health, and Proquest)	Database inception–November 2016	85 records	≥18 years	East Asia, Pacific region, Latin America, Caribbean, Europe, Central Asia, and (Middle East and North Africa) or Sub-Saharan Africa.	10	Mortality: CVD (0·47% (95% CI 0·34–0·61)).
	Chen et al. 2017 [103]	China’s Disease Surveillance Points system (DSPS)	January 2013–December 2015	272 Chinese cities	>5 years	China	10	Mortality: CVD (0.27% (95% posterior interval (PI), 0.18–0.36)), coronary heart disease (0.30% (95% PI, 0.19–0.40)), Stroke: 0.23% (95% PI, 0.13–0.34), cardiopulmonary disease (CPD) (17.55 (95% PI, 12.25–22.86)).
	Zhao et al. 2017 [104]	Meta-analysis (PubMed, and CNKI databases)	2007–2017	30 records	All ages	China	10	Mortality: CVD (0.68%, 95% confidence interval (CI): 0.39–0.97%).
	Amsalu et al. 2019 [105]	Beijing Public Health Information Center	January 2013–December 2017	460,938 admissions	18–64 years and ≥65 years	China, Beijing	10	Mortality: CVD (0.30, 95% CI: 0.20, 0.39%), CHD (0.34, 95% CI: 0.22 to 0.45%), Atrial Fibrillation (AF) (0.29, 95% CI, 0.03 to 0.55%).
	Tian et al. 2019 [106]	The urban employee basic medical insurance (UEBMI), urban resident basic medical insurance, and new rural cooperative medical scheme	January 2014–December 2017	8,834,533 hospital admissions	18–64 years, 65–74 years, and ≥75 years	China	10	CVD (0.26% (95% CI 0.17% to 0.35%)), Ischaemic heart disease (IHD) (0.31% (0.22% to 0.40%)), heart failure (0.27% (0.04% to 0.51%)), heart rhythm disturbances (HRD) (0.29% (0.12% to 0.46%)), ischaemic stroke (IS) (0.29% (0.18% to 0.40%).
	Wyatt et al. 2020 [107]	US Renal Data System (RDS)	2008–2014	361,568 patients	NA	U.S.	10	CVD (1.8%, 95% CI 0.4% to 3.2%), dysrhythmia, conduction disorder (4.8% (95% CI 2.3% to 7.4%)), and heart failure (3.7% (95% CI 1.4% to 6.0%).
	Qiu et al. 2020 [108]	Victim -crossover study of US New England Medicare participants	2000–2012	532,154 individuals	>64 years	U.S. New England- (states of Connecticut, Maine, Massachusetts, New Hampshire, Rhode Island, and Vermont)	10	CVD, acute myocardial infarction (AMI) (4.3% (95% CI: 2.2%, 6.4%)), and congestive heart failure (CHF) (3.9% (2.4%, 5.5%)), IS (2.6% (0.4%, 4.7%)).
	Dahlquist et al. 2020 [109]	Victim-crossover study of Stockholm	2012–2013 and 2016–2018	8899 individuals	75 years	Sweden-Stockholm	4.6	Acute AF.
	Farhadi et al. 2020 [110]	Meta-analysis (PubMed, Scopus, Web of Science, and Embase)	January 2000–January 2018	26 records	NA	NA	10	MI (relative risk (RR) = 1.02; 95% CI 1.01–1.03).
	Ren et al. 2020 [111]	Victim-crossover study of Shenyang, China	January 2014–December 2017	157,144 patients	0–30 years, 31–60 years, and >60 years	China-Shenyang Liaoning	10	CVD.
	Zhou et al. 2021 [112]	Taiyuan Center for Disease Control and Prevention	January 2013–October 2015	50,782 patients	>65 years	China-Taiyuan	10	Mortality: CVD (0.51% (95% CI: 0.08, 0.94)), IHD (1.01% (95% CI: 0.53, 1.50)), MI (1.08% (95% CI: 0.34, 1.83)).
	Yue et al. 2021 [113]	Meta-analysis (PubMed, Embase, the Cochrane library and Web of Science)	2015–2020	18 records	<65 years and >65 years	China, Sweden, Korea, U.S., Italy, Canada, Iran, Israel, Denmark	10	AF (1.01(95% CI 1.00–1.02) and 1.07 (1.04–1.10)).
	Kuzma et al. 2021 [114]	Victim-crossover study of Bialystok and Katowice in Poland, Europe	2008–2017	9046 patients	64–69 years	Europe-Poland	10	Incidence: STEMI (OR = 1.041, 95% CI = 1.020–1.073; P < 0.001, lag-1).
	Chen et al. 2021 [115]	Meta-analysis (PubMed, Embase, and Web of Science)	2006–2019	13 studies	<65 years and ≥65 years	North America, Europe, and Asia	10	AF (ER = 23.2%, 95% CI = −9.3–67.5), (ER = 0.6, 95% CI = −3.9–5.4), (ER = 2.3, 95% CI = 0.1–5.2).
Long-term PM_2.5_ exposure studies	Badaloni et al. 2017 [116]	Rome Longitudinal Study (RoLS)	October 2001–December 2010	1,249,108 individuals	30–44 years, 45–54 years, 55–64 years, 65–74 years, and >75 years	Italy, Rome	5	Mortality: IHD, CVD (hazard ratio (HR) = 1.05; 95% CI: 1.02–1.08), (HR = 1.06; 95% CI: 1.01–1.11).
	Jerrett et al. 2017 [117]	American Cancer Society Cancer Prevention Study II (CPS-II)	1982–2004	668,629 participants	≥30 years	U.S., Washington, DC, and Puerto Rico	10	Mortality: IHD.
	Kim et al. 2017 [118]	National Health Insurance Service–National Sample Cohort (NHIS-NSC)	2007–2013	1,025,340 individuals	≥18 years	Korea-Seoul	1	Mortality: cardiovascular event (CE) (1.36 (95% confidence interval, 1.29–1.43)) and Incidence: Stroke.
	Pinault et al. 2017 [119]	Canadian Census Health and Environment Cohort (CanCHEC)	2000–2008	2,448,500 participants	25–89	Canada	10	Mortality: IHD (HR = 1.16; 95% CI: 1.13–1.20).
	Pun et al. 2017 [120]	Medicare Beneficiaries	2000–2008	52.9 million participants	65–120	U.S.	10	Mortality: CVD (RR = 1.56, 95% CI: 1.55, 1.57)
	Qiu et al. 2017 [121]	Elderly Health Service of the Department of Health in Hong Kong	1998–2001	66,820 individuals	≥65 years	Hong Kong	10	Incident: Stroke (1.14 (95% CI 1.02–1.27).
	Stockfelt et al. 2017 [122]	PPS cohort and the GOT-MONICA cohort	1990–2011	10,350 participants	25–64 years and 64–75 years	Sweden-Gothenburg	5	IHD (HR: 1.24 95% CI: 0.98–1.59) and Incident: Stroke (HR: 1.48; 95% CI: 0.88–2.49).
	Turner et al. 2017 [123]	American Cancer Society Cancer Prevention Study-II	1999–2008	429,406 participants	<40 years and 40–80 years	U.S., Columbia, Puerto Rico etc.	11–15	Mortality: CV (relative excess risk due to interaction (RERI) = 0.10, attributal proportion (AP) = 0.05, synergy index (S) = 1.11).
	Yin et al. 2017 [124]	Disease Surveillance Points (DSPs), China	1990–1991	189,793 participants	≥40 years	China	10	Mortality: CVD (1.12 (1.10, 1.13)).
	Cakmak et al. 2018 [125]	Canadian Census Health and Environment Cohort (CanCHEC)	1991–2011	3.6 million participants	≥25 years	Canada	10	Mortality: IHD (1.13; 95% CI 1.08, 1.19).
	Gandini et al. 2018 [126]	Italian Longitudinal Study (ILS)	1999–2000	140,011 individuals	>35 years	Italy	10	AMI (1.15 (1.12–1.18)) and Incidence: Stroke.
	Loop et al. 2018 [127]	REasons for Geographic and Racial Differences in Stroke (REGARDS) cohort	2003–2007	17,126 participants	≥45 years	U.S. -Stroke Belt and Stroke Buckle	2.7	Mortality: CHD (0.94 (0.83–1.06)) and Non-fatal: AMI (0.85 (0.73–0.99)).
	Parker et al. 2018 [128]	National Health Interview Survey (NHIS)	1997 to 2009	657,238 participants	≥25 years	U. S.	10	Mortality: Heart disease ((HR, 1.16; 95% CI, 1.08–1.25)).
	Yitshak-Sade et al. 2018 [129]	Harvard School of Public Health Institutional Review Board	2001−2011	2,015,660 participants	≥65 years	U.S., New England	2.3	CVD (6.58% (5.90%; 7.26%)), and IS (0.82% (−0.68%; 2.35%)).
	Bai et al. 2019 [130]	Ontario Population Health and Environment Cohort (ONPHEC)	2001 to 2015	6,248,299 participants	35–85 years	Canada-Ontario	9.6	Mortality: CHF (1.05 (95% CI: 1.04–1.05)) and Incident: AMI (3%; (95% CI: 2–3%)).
	Danesh Yazdi et al. 2019 [131]	Medicare and Medicaid Services denominator file	2000–2012	11,084,660 individuals	≥65 years	Southeastern United States-Florida, Alabama, Mississippi, Georgia, North Carolina, South Carolina, and Tennessee	1	AMI and Stroke.
	Dirgawati et al. 2019 [132]	The Health in Men Study (HIMS)	Apr 1996–Jan 1999	12,203 participants	≥65years	Perth	5	Fatal: Stroke.
	Heritier et al. 2019 [133]	Swiss National Cohort (SNC)	Dec 2000–Dec 2008	7.28 million observations	>30 years	Switzerland	10	Mortality: AMI (1.034, 95% CI: 1.014–1.055).
	Huang et al. 2019 [134]	China-PAR	2000–2015	117,575 participants	<50 years >50 years	China	10	Incident: Stroke (13% (1.133, 1.09 to 1.17).
	Lim et al. 2019 [135]	National Institutes of Health–American Association for Retired Persons (NIH-AARP)	1995–2011	548,845 participants	50–71years	U.S. states (California, Florida, Louisiana, New Jersey, North Carolina, and Pennsylvania) and metropolitan areas (Atlanta, Georgia; Detroit, Michigan)	10	CVD (1.13; 95% CI, 1.08–1.18)), IHD ((HR, 1.16; 95% CI, 1.10–1.23)).
	Ljungman et al. 2019 [136]	Swedish cohorts (includes the Primary Prevention Study (PPS) and the Multinational Monitoring of Trends and Determinants in Cardiovascular Diseases (GOT-MONICA)	Jan 1990–Dec 2011	114,758 individuals	25–64 years	Sweden-Gothenburg, Stockholm, and Umea	1.94	Incident: IHD (6.5% (95% CI: −0.5−0.5, 14)).
	Pope et al. 2019 [137]	National Health Interview Surveys (NHIS)	1986–2014	1,599,329 participants	18–84 years	U.S.	10	Mortality: CP (1.24 (95% CI: 1.20, 1.29)) and (1.23 (95% CI: 1.17, 1.29)).
	Shin et al. 2019 [138]	Ontario Population Health and Environment Cohort (ONPHEC)	Apr 2001–Mar 2015	5,071,956 participants	35–85 years	Canada-Ontario	10	AF: HR (95% CI): 1.03 (1.01, 1.04) and Incidence: Stroke (HR (95% CI): 1.05 (1.03, 1.07)).
	Hayes et al. 2020 [139]	National Institutes of Health NIH-AARP	2000–2005	565,477 participants	50–71 years	U.S. states (California, Florida, Louisiana, New Jersey, North Carolina, and Pennsylvania) and urban areas (Atlanta, GA, and Detroit, MI,)	10	Mortality: IHD (HR 1.16; 95% CI 1.09–1.22) and Stroke (HR 1.14; CI 1.02–1.27).

## 6. PM_2.5_-Induced Risk of Cardiovascular Diseases

As discussed in Section 3, the pathophysiological mechanisms through which PM triggers cardiovascular events include the activation of oxidative stress/inflammation pathways, direct translocation into blood circulation, and autonomic imbalance. These alterations lead to subclinical CVDs (as shown in Figure 1) including atherosclerosis, coagulation, hypertension, myocardial remodeling, and thrombotic and non-thrombotic acute cardiovascular events such as heart failure, endothelial dysfunction, and arrhythmias [4,10], which are covered in the following sections.

### 6.1. Acute Coronary Syndrome and Myocardial Infarction

Acute coronary syndrome (ACS) is triggered by acute myocardial ischemia including unstable angina, ST-elevation MI (STEMI), and non-STEMI (NSTEMI) [140]. The link between exposure to PM_2.5_ and ACS development has been demonstrated in several systematic reviews and meta-analyses. Case-crossover reports have shown that patients with STEMI have an increased risk of PM-induced ACS [141,142]. Similarly, another time-stratified case-crossover study demonstrated the risk of acute coronary events upon short-term exposure to PM_2.5_, where excessive risk was observed in patients with preexisting coronary artery disease [143]. Furthermore, a PM_2.5_-related risk of MI was evidenced for STEMI events and not NSTEMI events, where a 10 μg/m^3^ elevation in concurrent-day PM_2.5_ was linked to an 8–15% increased risk of STEMI [143]. In a European cohort study that included 100,166 participants, each 5-μg/m^3^ increase in PM_2.5_ was linked to an 18% increase in nonfatal acute coronary events [83]. In a systematic review of 26 studies, PM_2.5_ exposure showed an increase in MI risk ranging from 5 to 17% per 10 µg/m^3^ increase [144]. In a meta-analysis of 34 studies, the associations between short-term exposure to PM_2.5_ and an increase in MI risk were evaluated [145]. In this study, a 2.5% increase in the risk of MI per 10 µg/m^3^ elevation was observed [145]. It is also evident from population-based cohort studies that long-term PM_2.5_ exposure substantially influences the survival of ACS and acute MI patients [146,147].

### 6.2. Arrhythmia

Cardiac arrhythmia is a group of conditions that trigger an abnormal heartbeat, and it is linked to a high incidence of CVD and mortality [148]. Several studies demonstrate that both high and low levels of PM_2.5_ exposure induce an increased risk of cardiac arrhythmias. A meta-analysis showed that short-term exposure to PM_2.5_ increases the incidence of arrhythmia hospitalization or mortality [149]. A multicenter longitudinal study assessed the effects of short-term PM_2.5_ exposure in high-risk patients with implantable cardioverter-defibrillators (ICD) or cardiac resynchronization therapy defibrillators (ICD-CRT) and demonstrated an association between the prevalence of ventricular tachycardia and ventricular fibrillation [150]. Long-term exposure to PM increases the risk of ventricular arrhythmia. Two population-based cohort studies from Canada and South Korea showed a link between PM_2.5_ and an increased incidence of atrial fibrillation (AF) [138,151]. Recently, in a large prospective cohort study, long-term exposure to PM_2.5_ exposure was associated with right-bundle branch block and bradycardia among middle-aged Koreans [152]. Previously, a meta-analysis of four observational studies suggested that PM_2.5_ was associated with a 0.89% increase in the AF risk per 10 µg/m^3^ elevation [153]. Recently, a meta-analysis of 18 studies also demonstrated that both short- and long-term exposures to PM_2.5_ had adverse effects on AF prevalence in the general population [113].

### 6.3. Cardiovascular Mortality

Several reports have suggested that both short- and long-term exposures to PM_2.5_ are linked to an increase in cardiovascular mortality. Time-series analyses of hourly, daily, and monthly variations in PM_2.5_ levels have identified the correlation between cardiovascular-related death and PM [4,15]. Low levels of daily exposure to PM_2.5_ showed an increment in the risk of 0.3–1.0% cardiovascular mortality per 10 μg/m^3^ increase in PM_2.5_. High levels of daily exposure to PM_2.5_ ranging from 39 to 177 µg/m^3^ was associated with a 0.35% excess risk of cardiovascular mortality per 10 μg/m^3^ increase in PM_2.5_ [154,155]. A meta-analysis identified that short-term exposure to PM_2.5_ (a daily increase of 10 μg/m^3^) was linked to a 0.84% surge in cardiovascular-related mortality [100]. Similarly, another quantitative systematic review demonstrated links between short-term exposure to nitrogen dioxide (daily increment of 10 μg/m^3^) and increases of 0.4–0.88% in deaths from CVD [156]. Long-term PM_2.5_ exposure (10 μg/m^3^ increment in annual PM concentration) was linked to an 11% increase in cardiovascular-related deaths [157]. In a large cohort study of long-term PM exposure in the American population, a 15% increase in deaths due to ischemic heart disease (IHD) per 10 μg/m^3^ increase in PM_2.5_ was observed [158]. Similarly, a Canadian national cohort study concluded that PM_2.5_ exposure at a very low concentration (mean, 8.7 μg/m^3^) increased cardiovascular-related mortality by 31% per 10 μg/m^3^ elevation [159]. Furthermore, several studies in China reinforce the link between high levels of PM_2.5_ exposure and increased cardiovascular-related mortality [155,160]. According to a large-scale prospective study involving women, long-term exposure to traffic-related pollutants was strongly correlated with cardiovascular-related mortality. An increase in sudden cardiac deaths of 38% was observed in a population living within 50 m of a major roadway compared with those living beyond 500 m [161].

### 6.4. Heart Failure and Ischemic Heart Disease

Congestive heart failure (CHF) is a chronic and progressive syndrome in which the heart muscle becomes incapable of pumping sufficient blood to meet the body’s demand for blood and oxygen. A systematic review of 35 studies reported that short-term PM_2.5_ exposure increased the relative risk of hospitalization due to heart failure or mortality by 2.1% per 10 μg/m^3^ elevation [85]. Similarly, a case-crossover study conducted in 26 large cities in China correlated short-term PM_2.5_ exposure to an increase in the risk of CHF hospitalization by 1.3% [162]. A large-scale population-based study in South Korea on healthy participants demonstrated that long-term exposure to PM_2.5_ increased with a hazard ratio of 1.44% per 1 μg/m^3^ [118]. In addition, pollutants of PM_2.5–10_ were also significantly associated with an increased risk of cardiovascular failure. However, there was no evidence to determine whether patients with ischemic heart failure or patients with non-ischemic heart failure are more susceptible to air pollution. 

### 6.5. Blood Pressure and Hypertension

A large body of evidence has shown that both short- and long-term exposure to PM_2.5_ could induce hypertension. Acute exposure to ambient PM_2.5_ (10 µg/m^3^) is associated with slight elevations in systolic and diastolic BP of 1–3 mmHg, while chronic exposure demonstrated elevated BP followed by the occurrence of incident hypertension [163,164]. In a systematic meta-analysis of 22 studies, long-term PM_2.5_ exposure showed a positive association between BP and an increase of 1.393 mmHg and 0.895 mmHg per 10 μg/m^3^ elevation for systolic and diastolic BP, respectively [165]. Biological factors such as age and sex may influence the PM_2.5_-related risk of hypertension. Previous studies have shown that youth may have a greater risk of PM-mediated hypertension than the elderly [166]. However, there are a few discrepancies in sex studies, where some show a higher influence of PM_2.5_ in men, while other reports demonstrate a higher influence in women [164,167,168,169]. Hence, studies that have shown sex differences in PM_2.5_ having impact on hypertension, are rare. Recently, a meta-analysis of 11 studies explored the association between long-term PM_2.5_ exposure and hypertension in women, where hazard ratios and odds ratios of 1.23 and 1.07, respectively, were obtained. Furthermore, subgroup analysis demonstrated that menopausal, non-white, and diabetic adults were more sensitive to PM_2.5_ exposure [170]. Randomized controlled studies on the effect of PM_2.5_ exposure on hypertension and vascular alterations have been well summarized elsewhere [26].

### 6.6. Vascular Dysfunction, Peripheral Arterial Disease, and Atherosclerosis

PM_2.5_, a major risk factor for vascular endothelial injury, is regarded as an early predictor of atherosclerosis [171,172,173]. Recently, Hu et al. identified that PM_2.5_ could induce endothelial injury and inflammation, causing endothelial dysfunction through NLRP3 inflammasome activation [174]. Both short- and long-term PM exposure have been associated with changes in endothelial function. Long-term exposure to PM_2.5_ in the Multi-Ethnic Study of Atherosclerosis (MESA) cohort [175] demonstrated relationships between PM exposure and changes in vascular function. Studies have shown that long-term exposure to PM is associated with reduced endothelial function by reducing the flow-mediated dilation of the brachial artery and vasoconstriction [175]. In addition to the above-mentioned reports, there are other studies that have shown an association between PM and endothelial dysfunction, which were well summarized in an earlier study [171]. However, studies on the association between PM and PAD are scarce. In a study among elderly populations, positive associations were observed between chronic and acute exposures to PM_2.5_ and increased PAD hospitalization rates of 0.26% and 4.4%, respectively [176]. In a cross-sectional study conducted in Germany with 4544 participants, a 5th- to 95th-percentile increment upon long-term exposure to PM_2.5_ was linked with high incidences of both low and high ankle-brachial indexes [177]. Several atherosclerosis biomarkers, including carotid intima-media thickness (CIMT), coronary artery calcium, and carotid plaques have been linked to air pollution, and a MESA-Air cohort study in the United States demonstrated that a 5-µg/m^3^ elevation in long-term PM_2.5_ exposure was linked to coronary artery calcification [178]. A meta-analysis including eight cross-sectional and three longitudinal epidemiological studies showed a positive correlation between CIMT and long-term exposure to PM_2.5_ [179]. Recently, PM_2.5_ was shown to promote the development of atherosclerotic plaques and plaque vulnerability through the TLR4 pathway [173].

### 6.7. Thrombosis and Coagulation

A large body of evidence supports the association between platelet function and PM exposure [4,180,181,182,183]. VTE, which includes deep vein thrombosis (DVT) and pulmonary embolism (PE), is the third leading vascular disease, affecting nearly 10 million people annually [184]. Although previous studies have linked PM exposure to VTE, studies on the relationship between PM_2.5_ exposure and the development of DVT and PE remain scarce. Long-term exposure studies on PM_2.5_ were linked to DVT and hypercoagulability [4]. Similarly, another study showed that long-term exposure to PM_2.5_ enhanced the risk of increased platelet counts in men and women by 17% and 14%, respectively, indicating adverse effects on blood coagulability [180]. Kloog et al. showed that long- and short-term effects of PM_2.5_ exposure on 453,413 DVT and 151,829 PE hospital admissions were associated with an increased risk of DVT (0.63% and 6.98% for short- and long-term exposure, respectively) and PE (0.38% and 2.67% for short- and long-term exposure, respectively) [185].

## 7. Strategies to Mitigate the Effects of PM_2.5_ on Cardiovascular Disease

**Figure 3:**
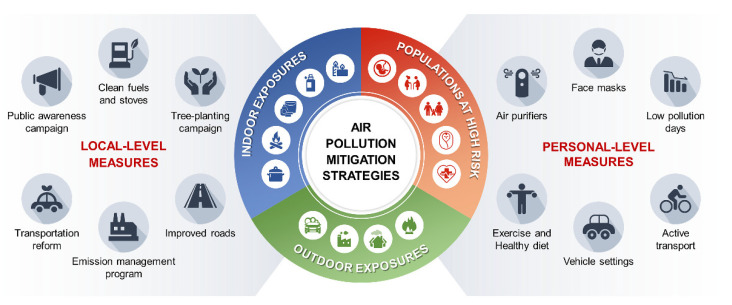
Local- and personal-level mitigation measures to reduce exposure to air pollution. The figure presents important elements related to reducing air pollution exposure and protecting respiratory and cardiovascular health. Sources contributing to both ambient and household air pollution are shown. Populations at high risk with exposure to air pollution include pregnant women, elderly individuals, children, newborns, and people with preexisting health conditions.

As of 2019, air pollution and climate change have been recognized by the WHO as top global environmental threats to human health. Recent refined modelling indicates that prior prediction models underestimated the health burden of air pollution and estimated that there were approximately nine million annual deaths due to air pollution globally [186]. More than 99% of deaths are due to household air pollution, and nearly 90% are due to ambient air pollution, occurring mostly in low- and middle-income countries, where people often burn solid fuels for cooking and heating. The sources of food derived from animals or plants also contribute to air pollution through ammonia, which is formed as a result of agricultural activities, animal farming, and food waste disposal. As awareness of air pollution increases worldwide, there is a need to provide evidence-based strategies to lessen its effects on health and the environment. Novel innovative and effective measures should be undertaken to tackle the formidable battle against air pollution at the global level. Both public and personal strategies play critical roles in mitigating exposure to air pollution (Figure 3).

### 7.1. Societal and Governmental Mitigation Strategies

Ambient air pollution causes several million premature deaths per year, mainly due to exposure to small particle sizes of ≤2.5 μm. In 2019, the State of Global Air reported that 40% of outdoor air pollution-related deaths were due to COPD, 30% of premature deaths were attributed to acute lower respiratory tract infections, 19% of deaths were attributed to lung cancer, 26% and 20% of deaths were attributed to stroke and IHD, respectively, 19% of deaths were linked to diabetes, and 20% were attributed to neonatal deaths. According to the WHO statistics, outdoor air pollution contributes to 4.2 million deaths annually, which are primarily due to cardiovascular and respiratory illnesses. To reduce outdoor air pollution, key policies that support air quality standards should be implemented such as energy-efficient housing, sustainable land use, agricultural incineration, better waste management, vehicle settings, emission management, improved road infrastructure, and transition to cleaner fuels, [187]. For example, the Clean Air Act program in the U.S. has estimated the prevention of more than 230,000 premature deaths associated with ambient PM by 2020. Such programs have lowered the levels of common air pollutants, including PM [135,188]. The air quality in the U.S. improved remarkably between 1990 and 2020, with the concentration of annual fine particles dropping by 41% [135]. A few major cities have already introduced policies aiming at reducing urban air pollution, such as bike sharing in Paris, congestion charging in London, and an environmental police force in Beijing.

To establish and enforce emission regulations, it is recommended that health care professionals and providers collaborate with governmental agencies and advocacy organizations. It is crucial for health care professionals to connect with patients through automated air pollution alert networks, including warnings through SMS, e-mail, or phones, such as the US EPA’s AirNow network [127,136]. Furthermore, individuals are encouraged to plan their activities through news feeds, websites, and mobile apps or applications to reduce their exposure to air pollution. Additionally, transitioning to cleaner fuels may be economically and logistically difficult for some countries, although others are making progress. For example, to provide clean cooking gas to 50 million poor Indian households, the Indian Ministry of Health allocated USD 1.5 billion [189]. While achieving carbon net zero is difficult, certain actions could be implemented including the replacement of current transportation systems with green or sustainable transportation, such as zero-emission vehicles, fitting vehicles with more effective filters and combustion engines, and introducing hybrid or electric vehicles. In places where there is a lack of electricity or gaseous fuels, solid fuels can be replaced with highly efficient stoves, improved fuels, ventilation, and education [190]. Moreover, protecting the integrity of ecosystems improves the health of communities worldwide. Therefore, the expansion of green land is necessary to prevent biodiversity loss, especially in highly urbanized and polluted countries [188]. Developing countries, such as China and India, are taking effective measures to expand the greening pattern to tackle two interconnected global problems, namely, air pollution and global warming, simultaneously [191].

### 7.2. Personal Mitigation Strategies

Individuals should be educated to reduce their exposure to traffic while commuting [192]. Commuting during peak hours should be avoided, particularly when using major roadways. Car travel should be accompanied by customs such as keeping the windows closed, maintaining the internal air circulation system, and utilizing a good air filtration system in cars [4]. On high-pollution days, people are advised to avoid or limit outdoor activities, keep windows closed, and use central air conditioning and air filtration systems [193]. Maintenance of a clean air circulation system has been shown to reduce the risk of cardiovascular-related hospitalization [194]. Under elevated ambient pollution levels, individuals are encouraged to perform exercise regimens indoors or in parks. While outdoors, individuals are encouraged to wear protective air filter-based equipment, such as personal face masks, to reduce air pollution exposure. Owing to the time-consuming implementation of policies to improve air quality, pedestrians and bicyclists choose personal masks or other devices to mitigate air pollution risks. Preliminary studies on the use of N95 masks by healthy individuals and individuals with heart disease in China have shown a reduction in BP, indicating an effective strategy for mitigating the effects of exposure to air pollution [195,196]. Similarly, another study from China showed low BP in healthy individuals who wore N95 masks [197]. These studies substantiate the use of particle-filtering masks to mitigate the short-term effects of urban air pollution on CVS caused by PM. Even though N95 masks filter out all but 5% of the particles, harmful gases remain unfiltered. To overcome this limitation, N95 masks combined with activated charcoal should be used to reduce exposure to harmful gases.

Recently, it has been suggested that glutathione and citrate attenuate cytotoxicity induced by urban PM in microglia [198]. The combination of glutathione and citrate reduces the toxicity of PM exposed to cells through mechanisms such as ROS scavenging, organic acid supplementation in the tricarboxylic acid cycle, and chelating Ca^2+^ ions. Further studies concerning the beneficial effects of glutathione and citrate are needed. A variety of dietary supplements, such as omega-3 polyunsaturated fatty acids, olive oil, and antioxidant vitamins have been shown to confer protection against autonomic and endothelial dysfunction and oxidative stress-induced reactions caused by air pollutants [199,200]. Individuals who are at a high risk of exposure to ambient air pollution could benefit from these dietary supplements. The most effective way to reduce indoor air pollution is to eliminate the burning of solid fuels. Access to cleaner fuels, such as liquefied petroleum gas, natural gas pipelines, and electric stoves, is critical for public health. Several studies have shown a positive association between the replacement of solid fuels with improved stoves and significant improvements in health outcomes [201,202,203]. Recently, two reviews have provided insights into personalized mitigation approaches for the reduction of exposure to air pollution in cardiovascular and respiratory health systems [154,204].

## 8. Conclusions and Future Perspectives

Outdoor and indoor pollution are recognized as major risk factors for premature mortality, morbidity, and decreased life expectancy, resulting in significant direct and indirect costs to society [3,205]. We have gained greater insights into the links between air pollution and cardiovascular-related morbidity and mortality through numerous epidemiological and experimental (in vitro and in vivo) findings. Improving air quality has led to a number of public health benefits, including improved longevity, lower mortality, and improved pathological studies [3]. Given the associations between health risks and air pollution, developed countries have implemented several initiatives to alleviate the effects of air pollution. According to the United Nations Environment Program, five cities, Paris, Seoul, New York, Bogota, and Accra, are taking innovative steps such as banning cars, implementing ultra-low emission zones, using green transportation, increasing green space, and switching to gas or electric stoves to achieve clean air [206]. However, the harmful effects of PM_2.5_ exposure on health necessitate more research efforts for gaining a deeper understanding of their pathogenic processes and development of useful tools for the prevention of primary and secondary diseases.

Accumulating evidence shows that PM affects not only the CVS but also the respiratory and central nervous systems. However, experimental and epidemiological evidence on PM_2.5_ is limited. In light of this, it is essential to gather more information about the dire effects of PM_2.5_ on sensitive populations. Moreover, it has been shown that the toxic effects of PM_2.5_ vary depending on its source and composition. For example, some components, such as BC, nitrates, and organic matter, and some metals, such as nickel and vanadium, are considered more dangerous than others. Furthermore, studies have shown that effects on cardiovascular health vary with the composition of PM_2.5_. For instance, some PM_2.5_ metals have shown toxic effects on health, such as increased levels of inflammatory blood markers and a greater risk of coronary events [207,208]. Variations in PM_2.5_ composition may also contribute to differences in findings among studies, and future research is needed to gain insights into the role of PM_2.5_ components in cardiovascular health risks. Hence, it is necessary to design several epidemiological and experimental studies to address the role of each component of PM_2.5_ and its associated adverse effects on health. Furthermore, more research efforts should focus on particles sized <100 nm, that is UFPs, which have been shown to exhibit more damaging effects on health. Because mitochondria are considered one of the primary targets for air pollutants, the further evaluation of mitochondrial epigenetics is necessary to thoroughly elucidate its biological mechanism and relevance in CVDs. Further investigations regarding the relationship between drug intake and air pollution are required. It would be interesting to evaluate whether widely prescribed drugs such as aspirin, β-blockers, and statins are associated with fewer cardiovascular events in comparison with non-prescribed groups who are equally exposed to high air pollution levels.

## Figures and Tables

**Table 1:** Key studies investigating the effects of PM_2.5_ on mitochondria in the cardiovascular system.

Study	Study Model, Pollutant and Year Published	Main Findings
Aung et al. [73]	In vitro model (human aortic endothelial cells (HAEC) exposed to both fine (PM_1.8_) and ultrafine particles (UFPs–PM_0.1_)), 2011	Gene responses involved in xenobiotic and oxidoreductase activities, inflammatory pathways, and transcription factors were affected.
Hu et al. [74]	In vitro model (HUVECs exposed to PM_2.5_), 2016	Decreased cell viability, increased LDH activity, increased ROS and MDA productions, inhibition of SOD activity, and increased levels of proinflammatory cytokines, cell adhesion molecules, and tissue factor. Upregulation of IL-6 dependent JAK1/STAT3 pathway.
Montiel-Dávalos et al. [75]	In vitro model (human umbilical vein endothelial cells (HUVEC) exposed to PM_2.5_), 2010	Increased production of reactive oxygen species (ROS) and nitric oxide (NO), and increased translocation of nuclear factor-kappa B (NF-κB) leading to apoptosis.
Sivakumar et al. [76]	In vitro model (H9c2 cardiomyocytes exposed to 100 µg/mL PM_2.5_), 2021	Augmented mitochondrial dysfunction and inactivation of PI3K/Akt signaling pathway (mitotoxicity).
Sivakumar et al. [77]	In vivo model (rat model of myocardial infarction (MI) exposed to PM_2.5_), 2022	Lowers mitochondrial endurance during cardiac recovery.
Sun et al. [78]	In vivo model (rats exposed to PM_2.5_ or filtered air for 10 weeks), 2008	Increase in mitochondrial superoxide production mediated by activation of Rho/ROCK pathway.
Wittkopp et al. [79]	Cohort study model (Elderly adults > 65 years with coronary artery disease—Measured hourly PM_2.5_), 2013	Toxic effects of air pollutants depend on the mitochondrial haplotype. Haplogroup H are more sensitive to air pollutants than haplogroup U.

## Data Availability

Not applicable.
